# Effects of corticotrophin releasing hormone (CRH) on cell viability and differentiation in the human BeWo choriocarcinoma cell line: a potential syncytialisation inducer distinct from cyclic adenosine monophosphate (cAMP)

**DOI:** 10.1186/1477-7827-11-30

**Published:** 2013-04-15

**Authors:** YuXia Chen, Megan Allars, Xin Pan, Kaushik Maiti, Giavanna Angeli, Roger Smith, Richard C Nicholson

**Affiliations:** 1 Department of Pathophysiology, Second Military Medical University, Shanghai, 200433, China; 2 Mothers & Babies Research Centre, Hunter Medical Research Institute, John Hunter Hospital, University of Newcastle, Newcastle, NSW 2305, Australia; 3 Department of Physiology, Second Military Medical University, Shanghai 200433, China

**Keywords:** Placenta, Cytotrophoblast, Differentiation, Syncytia, Syncytiotrophoblast, CRH, cAMP

## Abstract

**Background:**

Placental production of corticotrophin releasing hormone (CRH) rises exponentially as pregnancy progresses, and has been linked with the onset of normal and preterm labour. CRH is produced in syncytiotrophoblast cells and production is increased by glucocorticoids and cAMP. It remains unclear whether cAMP acts by inducing differentiation of cytotrophoblasts and/or through induction of syncytialisation. As CRH can stimulate cAMP pathways we have tested whether a feed-forward system may exist in placental cells during syncytialisation.

**Methods:**

The choriocarcinoma BeWo cell line was treated with cAMP, CRH or vehicle. Cell viability was determined by MTT assay, while apoptosis was analysed by DAPI staining and by FACS. Differentiation was measured by assaying message for hCG and ERVW-1 (syncytin1) by qRT-PCR, as well as the respective protein by ELISA. Fusion of BeWo cells was assessed by co-staining cell membrane and nuclei with CellMask and Hoechst 33342. CRHR1 and CRHR2 mRNA levels were measured by qRT-PCR.

**Results:**

We show that cAMP has an inductive effect on syncytialisation, as evidenced by induction of hCG secretion, by ERVW-1 mRNA expression and by formation of multinuclear cells. CRH mRNA expression was found to increase prior to the changes in the other syncytialisation markers. cAMP had an inhibitory effect on BeWo cell viability, but exogenous CRH did not. However, CRH did mimic the differentiation inducing effect of cAMP, suggesting a link between CRH and cAMP signalling in syncytialisation. We also found that treatment of BeWo cells with exogenous CRH resulted in elevated cellular CRHR1 levels.

**Conclusions:**

This study suggests a positive feed-forward role exists for CRH in trophoblast cell differentiation, which may underlie the exponential rise in CRH observed as gestation advances.

## Background

The differentiation of villous cytotrophoblast cells, the main cellular component of the placenta, is critical for a normal pregnancy as they mediate such steps as implantation, pregnancy hormone production, immune protection of the fetus, and delivery [1,2]. It is known that upon in vitro stimulation with cAMP-inducing agents, such as forskolin, cytotrophoblasts undergo fusion into syncytiotrophoblasts. A syncytiotrophoblast is defined as a giant cell with multiple nuclei sharing one cytoplasm, which expresses certain markers including ERVW-1 (syncytin 1) and human chorionic gonadotropin (hCG) [3,4]. The syncytial mass, as assessed by the number of nuclei, increases exponentially across gestation [5]. Many factors are involved in the process of syncytialisation in the placenta, including cAMP-dependent protein kinase A, various protein tyrosine kinases, protein tyrosine phosphatases, ERVW-1, and GCM1 [4,6]. While the list of players involved in cytotrophoblast differentiation is rapidly growing, the mechanisms remain far from clear.

Corticotrophin releasing hormone (CRH), one of the hypothalamic “stress” peptides, plays a pivotal role in mammalian survival and adaptation responses involving the activation of the hypothalamic pituitary adrenal (HPA) axis. Besides its presence in the central nervous system, CRH is also expressed in the human placenta [7]. Placental CRH becomes detectable in maternal plasma around 16~20 weeks gestation, and increases exponentially as pregnancy progresses towards term. The level of placental CRH in maternal circulation has been linked to the length of gestation [8-10]. Placental CRH appears to target multiple feto-maternal tissues, including the foetal adrenals, myometrial smooth muscle, placenta, placental vasculature and foetal membranes [11-13]. Through the various CRH receptor subtypes (CRHR1 and CRHR2) CRH plays diverse roles at different stages of pregnancy and labour. For example, CRH stimulates the foetal pituitary-adrenal axis, modulates placental vascular tone and endocrine function (especially prostaglandin generation), controls myometrial contractility ⁄ quiescence, and regulates trophoblast cell growth and invasion [10]. Nevertheless, there is little research literature available on a role for CRH in trophoblast cell differentiation.

We previously reported that stimulation by 8-Br-cAMP of BeWo cells cultured in normal foetal calf serum resulted in higher levels of hCG and ERVW-1 [14]. Moreover, other results from our laboratory have shown that CRH promoter activity is increased by 8-Br-cAMP in human placental cells [15], while others have shown that CRH can act via CRH-receptors to stimulate GNAS and increase cAMP production [16,17]. Therefore, in this paper we investigated potential roles of CRH on the viability and differentiation of human trophoblast BeWo cells.

## Methods

### Cell culture

The BeWo cell line, originally derived from a human trophoblast choriocarcinoma, was from the American Type Culture Collection. Cells were cultured in DMEM-F12 (Cat. D2906, Sigma-Aldrich, St Louis, MO, USA) supplemented with 10% foetal calf serum (FCS) (Cat. 12003C, SAFC Biosciences, Lenexa, Kansas, USA), L-glutamine (200 mM), HEPES (25 mM), glucose (25 mM), 1×Antibiotic-Antimycotic (Cat. 15240, Invitrogen, Mount Waverley, VIC, AU) and grown at 37°C in a 5% CO_2_ atmosphere. Once cells reached ~80% confluence they were harvested with trypsin and transferred into new tissue culture flasks with new media at a ratio of 1:3.

### MTT assay

Cells were seeded at 4×10^4^ cells per well in 24-well culture plates in triplicate, and allowed to attach and stabilize for 24 h. The medium was then changed to 1 ml per well of 10% charcoal stripped FCS (CCS) media (Cat. 12676, Invitrogen), followed by the administration of different doses of 8-Br-cAMP (Cat. B7880, Sigma-Aldrich) or CRH (C3042, Sigma-Aldrich) for a further 48 h. At indicated times, the viable cells were determined by MTT assay. Briefly, 100 μl of 5 mg/ml freshly made MTT solution was added to the cell media and incubated for 3 h at 37°C and dissolved with 750 μl DMSO. The absorbance at 570 nm and 630 nm (as background optical density) was then measured using 96-well plates (150 μl/well) with a FLUOstar OPTIMA microplate reader (BMG Labtech, Victoria, Australia).

### DAPI stain

Cells were seeded on coverslips pre-coated with 0.3% gelatine and set in 6-well plates at a density of 2 × 10^5^/well. The next day, cell culture media was changed to 10% CCS (charcoal stripped FCS) media and the cells were treated with 100 μM 8-Br-cAMP or 100 nM CRH for 72 h. Cells were then fixed with 2% paraformaldehyde solution for 15 min and permeabilized with 0.2% Triton-X-100 for 10 min. After washing with PBS, the cells were incubated with 2 μg/ml DAPI staining solution (0.1% Triton X-100, 2 mM MgCl_2_, 0.1 M NaCl, 100 mM 1,4-piperazine diethane sulfonic acid, 1 μg/ml DAPI pH 6.8) for 5 min. The slides were then washed, mounted and observed at 340–380 nm with Nikon Eclipse Ti fluorescent microscope (Nikon TI-SR, JAPAN).

### FACS analysis of apoptosis

BeWo cells were seeded in 6-well plates at a density of 2×10^5^/well. The next day, the culture media was changed to 10% CCS media with or without either 100 nM CRH or 100 μM 8-Br-cAMP for 72 hours. Cells were collected by trypsin digestion and rinsed in 1x binding buffer, before staining with 5 μl annexin-V-PE and 5 μl 7-ADD, using the PE Annexin V Apoptosis Detection Kit I (Cat. 559763 BD Biosciences, San Diego, CA, USA). After a 30 min incubation, samples were analysed by flow cytometry (BD Biosciences) to determine the proportion of apoptotic cells.

### Measurement of hCG secretion

BeWo cells were grown in 24-well plates, with the media replenished once with 10% CCS 24 h after seeding. Cells were then treated with 100 μM 8-Br-cAMP or 100 nM CRH for 72 hours. The supernatant was collected and kept at -80°C until assay, with the cell number determined by MTT assay. hCG quantification was conducted with a hCG ELISA kit (Cat. #0400, Alpha Diagnostic International, Texas, USA) according to the manufacturer’s instructions. The amount of hCG was normalized by the corresponding cell number and expressed as the fold of the untreated control.

### Cell fusion assay

To visualize the formation of cell fusion, 2×10^5^ BeWo cells were grown on 19-mm coverslips set in 6-well plates, and treated with either 100 μM 8-Br-cAMP or 100 nM CRH in 10% CCS media and cultured for 72 h. These cells were then incubated with 2 μg/ml CellMask Deep Red plasma membrane stain (Cat. C10045, Invitrogen) at 37°C for 5 min, followed by 5 μg/ml Hoechst 33342 (Cat. H1399, Invitrogen) for 10 min. After rinsing 3 times with PBS, cells were fixed with 4% paraformaldehyde for 15 min and mounted. Cell fusion was detected using a confocal microscope, and cell fusion events were scored when three or more nuclei shared the same cytoplasm, as we described previously [14].

### RT-quantitative PCR

Total RNA was extracted with RNeasy Mini kit (Cat. #74106, Qiagen, Doncaster, VIC, AU) and 1 μg of total RNA was used to synthesize first-strand cDNA using a Superscript Ш First-strand kit (Cat. #18080-051, Invitrogen), in accordance with the manufacturer’s instructions. qRT-PCR was performed using a 7500 real time system (Applied Biosystems, Warrington, WA1 4SR, UK) and the SYBR GREEN PCR Master Mix (Cat. 4309155, Applied Biosystems). The PCR reaction volume was 10 μl containing 2~3 μl of diluted cDNA and 0.2 μM of each primer. PCR conditions were set as an initial polymerase activation step for 2 min at 95°C, followed by 40 cycles of 15 sec at 94°C for template denaturation, 15 sec at 60°C for annealing and 20 sec at 72°C for extension and fluorescence measurement. In addition, each PCR reaction included a reverse transcription negative control to check for potential genomic DNA contamination. Possible reagent contamination was controlled by detection in a reaction mix without template. All samples were amplified in triplicates and the mean was used for qRT-PCR analysis. The ΔΔCT method [18] was applied to quantify the relative cDNA amount and ACTB was used as internal control. The primers used were sense 5^′^- GAAGGCCCTTCATAACCAATGA -3^′^,antisense 5^′^- GATATTTGGCTAAGGAGGTGATGTC -3^′^ for ERVW-1 (83 bp), sense 5^′^- CGGCATCGTCACCAACTG -3^′^,antisense 5^′^- AAGGTGTGGTGCCAGATTTTCT -3^′^ for ACTB (51 bp). Sense 5^′^-TCCCATCTCCCTGGATCTCAC-3^′^, antisense 5^′^-GTGAGCTTGCTGTGCTAACTGCT-3^′^ for CRH (85 bp), sense 5^′^- CGCATCCTCATGACCAAGCT -3^′^,antisense 5^′^- TCACAGCCTTCCTGTACTGAATG -3^′^ for CRHR1 (67 bp), sense 5^′^- TGCGGAGCATTCGCTGT -3^′^,antisense 5^′^- TTTCGCAGGATAAAGGTGGTG -3^′^ for CRHR2 (67 bp) [19].

### Statistical analysis

All experiments were performed in triplicate and all were repeated 3~5 independent times. The results are expressed as mean ± SD and the differences between groups were analysed using one-way ANOVA, with p < 0.05 being considered significant.

## Results

### Expression of mRNA for CRH and CRH receptors are induced by cAMP

**Figure 1:**
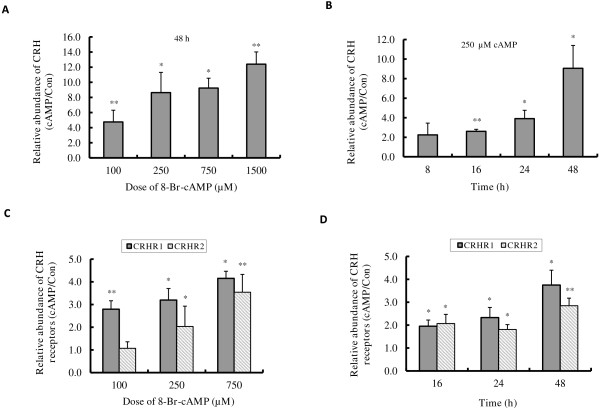
**8-Br-cAMP induced *CRH *and *CRHR *mRNA expression in BeWo cells. **BeWo cells were cultured in 10% CCS media and treated without or with 100~1500 μM 8-Br-cAMP for 8~48 h, the expressions of CRH (**A**, **B**) CRHR1 and CRHR2 (**C**, **D**) mRNA were determined by RT-quantitative PCR. Results shown are the induction fold relative to the control which was set at 1.0. Values are mean ± S.D. from 3 independent experiments. * p<0.05, ** p<0.01.

We have previously shown that 8-Br-cAMP increases CRH promoter activity in human primary placental cells [20]. In Figure 1 we show the effect of 8-Br-cAMP on the expression of endogenous mRNA encoding components of the CRH signalling pathway in BeWo cells.

BeWo cells were cultured in 10% Foetal Calf Serum (FCS) media and treated with 0~1500 μM 8-Br-cAMP for 48 h (Figure 1A) or 250 μM 8-Br-cAMP for 8~48 h (Figure 1B), and the level of CRH mRNA was quantified by qRT-PCR. Exposure to 8-Br-cAMP resulted in a dose- and time- dependent increase in CRH mRNA; 2.2- fold at 8 h and 8.6- fold at 48 h for the 250 μM treatment (Figure 1A, B). We also found that the mRNA levels for CRH receptors (CRHR1 and CRHR2) were also stimulated by 8-Br-cAMP treatment in a dose dependent manner (Figure 1C). Following exposure to 250 μM 8-Br-cAMP, the increases of CRHR1 mRNA were ~2- fold at 16 h and ~4- fold at 48 h, whereas CRHR2 mRNA expression was fairly constant from 16 h to 48 h, although there is a ~2- fold increase observed compared to non-treatment control (Figure 1D).

### cAMP but not CRH stimulates apoptosis in BeWo cells

**Figure 2:**
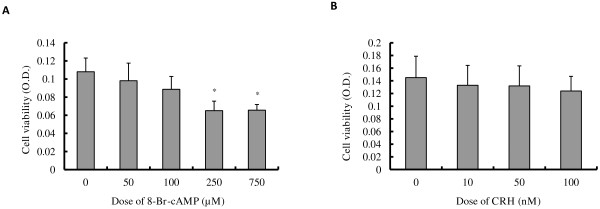
**Effect of 8-Br-cAMP and CRH on cell viability. **BeWo cells were cultured in 10% CCS media and treated with 0~750 μM 8-Br-cAMP (**A**) or 0~100 nM CRH (**B**) for 48 h, and the viable cell number was determined by MTT assay. All experiments were performed 4 times and the values plotted are mean ± S.D. * p<0.05, compared to control.

Previously published evidence has shown that BeWo cell viability is decreased by forskolin through increased cAMP [21], and that CRH can induce cell apoptosis in several cell lines including the rat pheochromocytoma cell line PC12, human lymphocytes and extravillous trophoblast cells [22-24]. Therefore, we examined the potential role of CRH on the cAMP-mediated effect on cell survival of BeWo cells. We found that when BeWo cells were treated with 100~750 μM 8-Br-cAMP for 48 h, there were more detached cells floating in the culture media compared to the control cells. MTT assay showed that cell viability decreased with increasing dose of 8-Br-cAMP (Figure 2A), with ~18% decrease in viability at 100 μM 8-Br-cAMP and significantly less viability (~39% decrease in viability at 750 μM) at higher doses of 8-Br-cAMP. In contrast, a 48 h incubation with 10~100 nM CRH (Figure 2B) resulted in very little reduction of cell number (~15% inhibition at 100 nM) without any sign of cell death.

**Figure 3:**
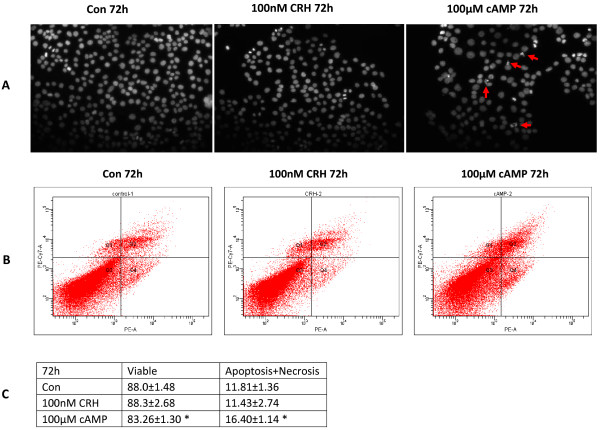
**Effect of 8-Br-cAMP and CRH on cell apoptosis. **BeWo cells were cultured in 10% CCS media and treated without or with 100 μM 8-Br-cAMP or 100 nM CRH for 72 h. (**A**) DAPI stain of apoptotic nuclei. Cells were fixed and nuclear morphology were observed by staining with DAPI and visualized by fluorescence microscopy (20×). Data shown are representative results from 3 independent experiments. Arrows shown are the condensed nuclei or nuclear fragments described in the text. (**B**) FACS analysis of apoptotic portion. Cells were collected, stained by Annexin V/7-ADD and measured by flow cytometry. Data shown is representative of FACS analysis. (**C**) Statistic analysis of apoptotic cells based on Figure 3B. Data were obtained from three independent experiments. *p<0.05.

Based on these results, and because there is no significant inhibition of cell viability, we chose 100 μM cAMP and 100 nM CRH as the treatment dosage for further experiments. Using DAPI staining, we investigated the morphological changes in the nuclei of BeWo cells following 8-Br-cAMP or CRH treatment. Figure 3A shows that no apoptotic nuclei are apparent when BeWo cells are cultured for 72 h with 100 nM CRH, whereas apoptotic nuclei debris is clearly visible in cells cultured with 100 μM cAMP for 72 h. Flow cytometry results (Figure 3B, C) also revealed that the apoptotic and necrotic portions were significantly increased by 72 h of 100 μM cAMP treatment (16.40±1.14) as compared to the control (11.81±1.36). However, there was no change between the control and 100 nM CRH treatment samples (Figure 3B, C).

### CRH up-regulates CRHR1 and CRHR2 mRNA expression

**Figure 4:**
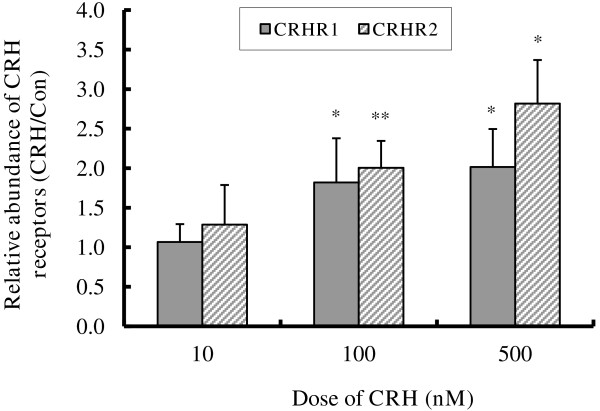
**CRH induced *CRHR *mRNA expression in BeWo cells. **BeWo cells were cultured in 10% CCS media and treated without or with 10~500 nM CRH for 48 h, the expressions of CRHR1 and CRHR2 mRNA were determined by RT-quantitative PCR. Results shown are the induction fold relative to the control which was set at 1.0. Values are mean ± S.D. from 3 independent experiments. * p<0.05, ** p<0.01.

Since CRH receptors are crucial in mediating CRH bioactivities, the lack of cell apoptosis in response to CRH prompted us to examine whether down-regulation of CRH receptors was occurring due to extended CRH exposure. In Figure 4, BeWo cells were treated with 0~500 nM CRH for 48 h in 10% charcoal stripped FCS (CCS) media, and the levels of CRHR1 and CRHR2 mRNA were quantified by qRT-PCR. We observed that CRH up-regulated the expression of both CRHR1 and CRHR2 in a dose dependent manner. Although 10 nM CRH had no significant effect on the expression of the CRH receptors, CRHR1 mRNA expression increased by 1.8-fold at 100 nM and approximately 2-fold at 500 nM CRH treatment. Similarly, CRHR2 increased by about 2-fold and 2.8-fold at 100 nM and 500 nM CRH respectively (Figure 4). This indicates that the CRH signalling pathway is intact and that the potential for a positive feed-forward pathway exists in BeWo cells.

### Cell differentiation of BeWo cells was promoted by both 8-Br-cAMP and CRH

**Figure 5:**
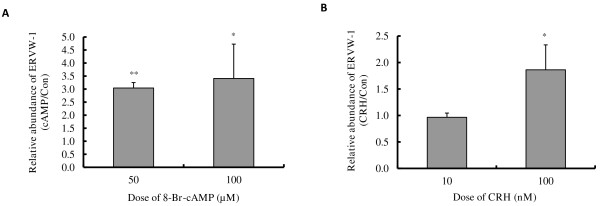
**Effect of 8-Br-cAMP and *CRH *on *ERVW*-*1 *mRNA expression. **BeWo cells were cultured in 10% CCS media and treated with or without 50~100 μM 8-Br-cAMP (**A**) or 10~100 nM CRH (**B**) for 48 h, the expression of ERVW-*1 *mRNA was determined by RT-quantitative PCR. Results shown are the induction fold relative to the control which was set at 1.0. Values are mean ± S.D. from 3 independent experiments. * p<0.05.

**Figure 6:**
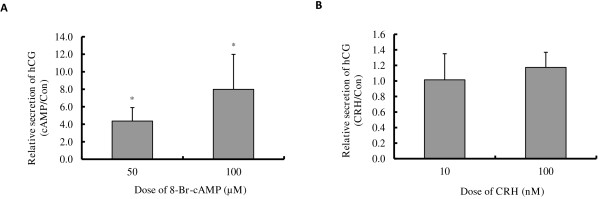
**Effect of 8-Br-cAMP and CRH on hCG secretion. **BeWo cells were cultured in 10% CCS media and treated with or without 50~100 μM 8-Br-cAMP (**A**) or 10~100 nM CRH (**B**) for 72 h. The amount of hCG in the media was measured by ELISA assay. The respective cell number was determined by MTT assay. The relative amount of hCG secretion was normalized by the cell number. Results shown are the induction fold relative to the control which was set at 1.0. Values are mean ± S.D. from 3 independent experiments. * p<0.05.

**Figure 7:**
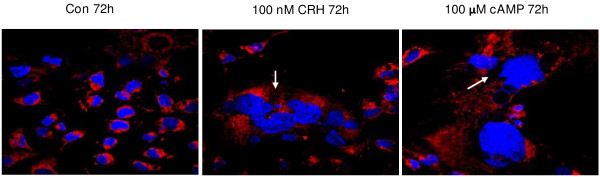
**Effect of 8-Br-cAMP and CRH on cell fusion in BeWo cells. **BeWo cells were cultured in 10% CCS media and treated without (**Con**) or with 100 nM CRH or 100 μM 8-Br-cAMP for 72 h. Cells were stained with CellMask plasma membrane stain (red) and Hochest 33342 nuclear stain (blue), and visualized by confocal microscope (100×). Arrows indicate position of syncytium. The results are representative of 3 independent experiments.

As cAMP is a key component of the signalling pathway that promotes cytotrophoblast cell fusion, we queried whether CRH has any effect on advancing BeWo cell differentiation. The effects of 8-Br-cAMP and CRH on the molecular markers of cytotrophoblast differentiation, ERVW-1 mRNA expression (Figure 5A, B) and hCG secretion (Figure 6A, B), were determined by qRT-PCR and ELISA assay respectively. 8-Br-cAMP treatment had no detectable effect on the expression of ERVW-1 within 24 h (data not shown), but at 48 hours following 50 or 100 μM 8-Br-cAMP treatment ERVW-1 mRNA expression was induced by 3.04- and 3.4- fold (Figure 5A). 10 nM CRH did not induce ERVW-1 mRNA expression while 100 nM CRH increased it by 1.87- fold (Figure 5B). As determined by ELISA, hCG secreted into the culture media increased by 4.4- and 8.0- fold (Figure 6A) when BeWo cells were treated with 50 or 100 μM 8-Br-cAMP treatment for 72 h respectively. However, 10~100 nM CRH had no effect on hCG secretion (Figure 6B). Fusion of BeWo cells was assessed by co-staining cell membrane and nuclei with CellMask and Hoechst 33342. Cell fusion, as defined by the presence of multinucleated cells, was seldom seen in control cells, but multinucleated cells containing more than four nuclei could always be seen in cell cultures treated with either 100 μM cAMP or 100 nM CRH for 72 h (Figure 7). This indicates that both cAMP and CRH are effective in promoting cytotrophoblast cell differentiation.

## Discussion

CRH is known to activate the cAMP/PKA (protein kinase A) pathway via its receptors, CRHR1 and CRHR2, leading to phosphorylation of the cAMP response element binding protein (CREB1) [25]. As a transcription factor, CREB1 binds to a cAMP response element (CRE) sequence in the promoter of target genes, regulating the transcription of those genes [26,27]. CRH has been studied as one of those target genes containing a CRE in the promoter, and the CRE has been shown to be crucial for CRH gene expression [28-30]. Furthermore, CRH and its receptors (CRHR1/R2) are expressed in placental tissues [9,31] and CRH can bind to syncytiotrophoblast membranes [32]. So, the induction of CRH by cAMP hints that CRH may play a feedback role in cAMP mediated effects in placental trophoblasts.

Using BeWo choriocarcinoma cells as a well-established cellular model of placental trophoblast syncytialisation we showed that, in the presence of serum, 8-Br-cAMP significantly induces cell apoptosis and promotes differentiation. Our previous data also revealed that 8-Br-cAMP had less effect on BeWo cell viability and syncytialisation if cells were cultured in 10% charcoal stripped media [14], since charcoal stripping would remove other factors such as steroids, leading us to imply that CRH signal pathways may play a role in cAMP-mediated cell apoptosis and differentiation. The ability of CRH to regulate cell apoptosis in several cells of different origin has been reported [33,34], and CRH has been shown to induce FASLG (Fas ligand) production and apoptosis in the rat pheochromocytoma cell line PC12 via activation of MAPK (p38 mitogen-activated protein kinase) [22]. This prompted us to further propose that although CRH is produced by differentiated trophoblasts it may also play a role in regulating trophoblast viability, thereby helping to keep the syncytiotrophoblast pool in renewal. However, our experiments reveal that although CRH induces cell apoptosis in some cell lines it has no significant effect on BeWo cell viability, according to DAPI stain and FACS analysis. This also suggests that any effect that 8-Br-cAMP has on BeWo cell viability is mediated through a signal pathway other than a CRH pathway. Indeed, these results indicate that while CRH may activate GNAS pathways, cAMP may activate additional compartments of cAMP mediated signalling, or CRH may activate additional pathways that antagonise apoptosis effects mediated by cAMP. Nevertheless, the existence of CRH receptors in BeWo cells suggests that CRH could be involved in another bioactivity, such as modulation of differentiation or syncytialisation of trophoblast cells. Consistent with this idea, we demonstrated that CRH can induce cell differentiation, as evidenced by the induction of ERVW-1 and by cell fusion. For trophoblast cells, the widely accepted marker of differentiation is a combination of a biochemical index (induction of hCG and ERVW-1 proteins) and a morphological index (formation of cell fusion), with the latter being more convincing. Recently, the reliability of hCG as a trophoblast differentiation marker has been called into question, due to dissociation of the two differentiation indexes. We show here additional evidence that biochemical and morphological differentiation can be dissociated, since CRH facilitates cell fusion without any induction of hCG secretion. Also, in primary human trophoblasts, CRH stimulation induced ERVW-1 expression, without any induction of hCG secretion [35]. Similar dissociations have been reported in villous cytotrophoblast cells cultured in the absence of serum [36] and in JEG3 cells stimulated by forskolin, where an induction of hCG occurred without any evidence of cell fusion [21]. Recently, it was reported that the PKA inhibitor, H89, can reverse forskolin-induced BeWo cell fusion without altering forskolin-induced hCG secretion [37]. Therefore, hCG protein expression may not necessarily be linked to syncytial fusion. This study adds to the growing evidence that differentiation and fusion are related but distinct events [38]. However, the secretion of hCG can be enhanced by 8-Br-cAMP but not by CRH, suggesting that CRH and 8-Br-cAMP may regulate cell fusion through different signal pathways. Indeed, others have shown that charcoal scavenged hormones, such as estradiol, glucocorticoids and hCG, play roles in trophoblast differentiation at different stages of pregnancy [39].

We recently published evidence of a coherent relationship between the induction by cAMP of apoptosis and syncytial differentiation in BeWo cells. In cells cultured in media containing serum, a large increase in FASLG/FAS ratio and the occurrence of cell apoptosis coincided with a greater induction of ERVW-1 and hCG and the formation of syncytia. In contrast, in cells cultured in media lacking serum, the lower increase in FASLG/FAS ratio with no apparent cell apoptosis is in agreement with the lower induction of ERVW-1 and hCG with no sign of cell fusion [14]. This relationship has prompted us to propose that the cytotrophoblastic syncytialisation process involves an apoptotic event. Clearly, the data in this paper shows that 8-Br-cAMP induces both cell apoptosis as well as cell fusion whereas CRH has no effect on apoptosis but can still promote cell fusion, indicating that syncytialisation is a process that can occur either with or without cell apoptosis. 8-Br-cAMP and CRH both elevate intracellular cAMP, with 8-BR-cAMP resulting in a sustained intracellular cAMP elevation resistant to phosphodiesterases, whereas elevations via cAMP are transient and compartmentalized and, as in other studies, this could determine whether elevating cAMP is pro- or anti- apoptotic [40]. Thus, whether the syncytialisation process correlates with an apoptosis program or not is far more complex than previously realised, and may depend on either, or both, the inducer and the cell model used. Furthermore, we found that the expression of CRHR1 and CRHR2 can be up regulated by 8-Br-cAMP and also by CRH itself. This latter finding allows the possibility that CRH and the CRH-receptors are involved in a positive feed-forward system that may underlie the exponential rise in CRH observed across pregnancy that leads to delivery. This requires further investigation but, if correct, suggests the timing of birth in humans is an event determined by a placental maturation program.

## Conclusions

Our data provides, for the first time, evidence that there may exist a positive feed-forward mechanism in the CRH/CRHR/cAMP/PKA/CREB1 signal pathway, and that this occurs at several different levels. The positive communication existing with CRH and its receptors has the potential to magnify CRH bioactivities by, for example, promotion of trophoblast cell syncytialisation. Since placental CRH expression in trophoblast cells begins when the cells differentiate toward syncytialisation, the positive feed-forward and promotion of syncytialisation by CRH could explain the exponential increase of CRH detected in maternal plasma as pregnancy progresses towards term, particularly as syncytial nuclear numbers increase exponentially across gestation [5]. This may provide the mechanism that links maturation of the placental structure to the timing of birth.

## Abbreviations

8-Br-cAMP: 8-bromo- cyclic adenosine monophosphate; cAMP: Cyclic adenosine monophosphate; CRE: cAMP response element; CREB: cAMP response element binding protein; CRH: Corticotrophin releasing hormone; CRHR: Corticotrophin releasing hormone receptor; ELISA: Enzyme linked immunosorbent assay; FACS: Fluorescence activated cell sorting; hCG: Human chorionic gonadotrophin; PCR: Polymerase chain reaction; PKA: Protein kinase A; qRT-PCR: Quantitative real time reverse transcriptase polymerase chain reaction.

## Competing interests

The authors declare that they have no competing interests.

## Authors’ contributions

YXC initiated and designed the project, performed most of the experiments and wrote the manuscript. MA helped with conducting and analysing RT-quantitative PCR. KM helped in design and analysis of the cell fusion assay. GA helped in performing cell culture and in manuscript preparation. XP helped with ELISA assays and RT-quantitative PCR. RS provided lab space and contributed to manuscript preparation. RCN contributed to the design of the project, data analysis and manuscript preparation. All authors read and approved the final manuscript version.
